# The impact of risk perceptions and belief in conspiracy theories on COVID-19 pandemic-related behaviours

**DOI:** 10.1371/journal.pone.0263716

**Published:** 2022-02-08

**Authors:** Jack P. Hughes, Alexandros Efstratiou, Sara R. Komer, Lilli A. Baxter, Milica Vasiljevic, Ana C. Leite

**Affiliations:** 1 Department of Psychology, Durham University, Durham, United Kingdom; 2 Department of Computer Science, University College London, London, United Kingdom; University of Haifa, ISRAEL

## Abstract

Throughout the COVID-19 pandemic, conspiracy theories about the virus spread rapidly, and whilst governments across the globe put in place different restrictions and guidelines to contain the pandemic, these were not universally adhered to. This research examined the association between pandemic related risk perceptions, belief in conspiracy theories, and compliance with COVID-19 public guidelines amongst a UK sample (*n* = 368). Participants rated their level of concern for a series of potential risks during the pandemic (to the economy, personal health, freedom, media integrity and health risk to others). Participants also rated their level of belief in different conspiracy theories and self-reported their behaviour during the first UK lockdown. Mediational analyses showed that stronger belief in conspiracy theories was associated with perceptions of lower risk to health and higher risk to the economy and freedom, which in turn were associated with lower compliance with COVID-19 related governmental guidelines. Perception of information transparency risks did not mediate the association between belief in conspiracy theories and compliant behaviours. These results highlight the key role that risk perception may play in translating belief in conspiracy theories into low compliance with governmental COVID-19 related guidelines. Our findings suggest new patterns with respect to the relationship between conspiracy theory adherence and salience of different risk perceptions amidst the pandemic, which could have implications for the development of public health messaging and communication interventions.

## Introduction

The COVID-19 pandemic created many challenges globally, with over 225,000,000 cases across 194 countries [1]. The way individuals have responded to the pandemic, however, seems to have differed widely. Anecdotal evidence suggests that levels of compliance with governmental restrictions and guidelines varied greatly, with some people taking greater risks than others [2–5]. It is possible that varying behaviours during the pandemic may be related to individuals’ differing perceptions of risk. In this paper, we examined the interplay between individual appraisals of risk, levels of conspiracy theory belief, and behaviour during the pandemic.

Since the COVID-19 virus swept across the world, a large number of related conspiracy theories have gained traction [6]. Conspiracies range from presenting COVID-19 as a hoax to connecting it to unrelated events such as the increase in 5G technology usage [7]. These views, and other pandemic-related conspiracy theories, have been found to negatively correlate with several behaviours, such as handwashing, keeping physical distance, and hoarding [8]. The effect of belief in conspiracy theories on reducing compliance with government guidelines and health regulations has been demonstrated in different contexts in England [9], the US [10] and Poland [11]. Recent research has also shown that perceptions of the individual health risk of COVID-19 and the fear of illness positively correlate with willingness to comply with government regulations [12, 13]. Not all risk perceptions and fears are based around health, however. Members of the public may also fear the economic damage or the loss of individual freedoms that may occur due to the pandemic. These varying risk perceptions may cause individuals to behave in vastly different ways depending on what they worry about or fear. The way in which the virus’ level of threat has been framed has also led to disagreement, with many people referring to the small mortality rate (framed percentage-wise), approximately 0.02% globally, and others referring to the large number of people who died (framed in absolute numbers), over 5.3 million as of December 2021 [1]. It is plausible that perceptions about the level and type of threat of the virus, and the pandemic more generally, may influence individuals’ behaviours.

This study examined whether belief in conspiracy theories and differential perceptions of risk were associated with individual behaviours during the pandemic.

### Conspiracy theories

Perceptions of risk may amplify or work in tandem with the effects of belief in conspiracy theories in predicting adherence to lockdown measures. In the previous 2009 influenza pandemic, conspiracies also rapidly gained traction, and they involved similar beliefs such as the virus being created in a lab, government control, and international attacks [14]. Events that have a large impact may lead people to seek explanations that are significant in a similar scale [15]. This is commonly referred to as the ‘major event-major cause’ heuristic [16] and has been shown to influence to what extent we might believe a conspiracy theory if we feel it offers a more ‘proportional’ explanation to an event. For example, McCauley and Jacques [16] found that, when given a scenario whereby a fictitious president is a victim of an assassination attempt, participants were more likely to believe the attempt was part of a larger conspiracy if the president was successfully assassinated than if the president survived. This finding was later replicated by Leman and Cinnirella [17] and offers evidence that the severity of an outcome influences how likely people are to believe it is part of a larger malicious plot. In the context of COVID-19, the potentially humble inception of the virus via natural means may not seem proportionally significant enough to explain the far-reaching, global, and severe impact of the pandemic, which may fuel conspiracy theories.

Previous research has found that exposure to conspiracies that are commonplace online, such as climate change denial or vaccination myths, are associated with mistrust in governments [18] and can influence critical medical, political, and environmental behaviours [19, 20]. The more time individuals spend on the internet, the less they trust the government and comply with regulations [21]. Therefore, exposure and subsequent belief in COVID-19 related conspiracies could directly impact people’s behaviour in response to government guidelines and restrictions. Although conspiracy theorists often receive their information from social media sources, which perpetuate conspiracies [22], they typically show skepticism towards mainstream media which offers more scientifically sound resources [23]. Some recent work has also shown that COVID-19 conspiracies predicted a decrease in preventive actions and vaccination intentions [24, 25]. Furthermore, belief in conspiratorial governments during the pandemic has been found to contribute to less frequent preventive action such as washing one’s hands and social distancing [11], while other sources claim that higher trust in the government correlated with lower risk perceptions which predicted less preventive measures [26]. Looking at previous outbreaks, such as H1N1, citizens were more likely to obtain appropriate supplies and comply with preventive measures when they trusted their local governments to handle emergencies instead of looking towards conspiracy theories [27]. Therefore, conspiracy belief may be partly driven by a lack of trust in local government [28]. There is still, however, uncertainty about the impact of risk perceptions on the behaviours of citizens during COVID-19 and what the perceived risks that promote compliant behaviour are. The extant literature on the relationship between government distrust and belief in conspiracy theories points to the idea that government guidelines are not viewed as an attempt to protect the public by conspiracy theory advocates, but rather, an attempt to restrict individual freedoms and liberties [29]. Given previous evidence relating conspiracy theory belief to perceptions of government manipulation and plotting [30], it is likely that belief in conspiracy theories may be associated with freedom-suppression related risks.

Furthermore, belief in different types of COVID-19 conspiracy theories have been associated with different responses and behaviours. For example, people who endorsed conspiracy theories that portrayed COVID-19 as a hoax were less likely to engage in behaviours such as social distancing and handwashing whereas conspiracy theory advocates who believed COVID-19 was created by an evil source were more likely to participate in behaviours such as hoarding [8]. Conspiracy theory advocates were also less likely to perceive a major health-related risk to COVID-19 based on the circulating theory that the virus does not exist or is not dangerous to their personal health [31]. However, when conspiracy theory advocates perceived COVID-19 as a life-threatening health risk, they were more likely to try and protect themselves by complying with government guidelines [32]. Since conspiracy theory advocates operate from a self-centered perspective, such as protecting themselves [33] or feeling that they possess unique information [34], belief in conspiracy theories may correlate negatively with the perceived risks posed to other people. Whether this divergence in behavioural actions is due to belief in conspiracy theories influencing people’s risk perceptions requires further investigation. Furthermore, due to the fact that conspiracies are bred out of trying to explain uncertainty [3, 35], belief in conspiracy theories could positively predict uncertainties related to economic dimensions, but further investigation is required.

### Risk perceptions

People’s individual risk perceptions are influenced by factors including their level of willingness to undertake the risk, knowledge about the risk, trust in those managing the risk, and the visibility of the threat [36]. A recent study found that, during the COVID-19 pandemic, the level of concern about individual health varied amongst regions as well as cultures and was highest in the UK compared to other sampled countries [26]. Additionally, social media has been found to strongly impact risk perception by significantly covering stories on novel emerging diseases such as H1N1 compared to viruses that have been prevalent for a long time and are potentially more harmful [37, 38]. Since more unknowns exist with novel diseases in the early stages of emergence, individuals often turn to the media for explanations and evaluation of risk levels [39], and the levels of COVID-19 health risk perceptions may be expounded upon by the risk perception of misinformation [40]. For example, although U.S. citizens generally agreed on the risk of COVID-19 on health, the economy, and personal finances, political parties drastically disagreed on whether the media exaggerated and misrepresented the novel disease’s risk level [41]. When messages about risk are not understood by the groups at risk, messaging is conflicting, or the general source lacks trustworthiness, this will often lead to a lack of preparation to protect from the risks [42]. On the other hand, since the media often rapidly picks up news about emerging diseases, this may drastically increase risk perception and lead to ineffective precautionary methods [42]. Risk perceptions are therefore complex to manage and understand, especially during times of crisis.

Understanding the effect of risk perception on people’s behaviour is of vital importance. Risk perceptions focusing around individuals’ health have been shown to be influential in terms of people’s adherence to safety measures at work [43], participating in animal rescue behaviours in bushfires [44], willingness to move houses to avoid flooding [45], and adoption of preventive behaviours during the pandemic [26]. To further test whether risk perceptions are the underlying construct that is associated with compliant behaviours and how the differing categories of risk concerns influence pandemic responses, we identified a series of variables that could prove integral to acting compliantly during the COVID-19 pandemic. For example, a recent study found that risk perception towards oneself did not result in vaccine intention whereas risk perception awareness of others did [46]. Additionally, although adults in the U.S. displayed a higher perceived risk for infection, they perceived less of a risk for mental health throughout the beginning of the pandemic [47]. Therefore, it is important to examine various risks instead of solely focusing on infection risk to fully understand what drives behaviour during the pandemic. We reasoned that economic, health, and media risks are all unique concerns that need to be studied.

The perception of risk to personal health has been shown to predict adherence to social distancing in Qatar [48] and improve protective behaviours in the U.S. [49] during the COVID-19 pandemic. In one study carried out in the U.S., an increase in perception of risk correlated with an uptick in engaging in compliant behaviours such as social distancing and hand washing. Furthermore, this study found that individuals who perceived low levels of risk during the COVID-19 pandemic did not adopt compliant behaviours [50]. However, mixed findings have also been reported. One set of studies demonstrated a strong interaction between risk perceptions and actions in opposition to general protective behaviours (such as with respect to cycling) [51–53], while another found no relationship between risk perception and general protective behaviour [54–56]. The theory of risk aversion demonstrates how people will act irrationally in order to escape perceived risks, regardless of whether those risks are present or objectively salient [57, 58]. Different individuals may be more attuned to different kinds of risks and therefore have particular risk perceptions which may shape their behaviour. In practice, this could translate into people who are more focused on economic risks to circumvent lockdown regulations, or people who overestimate health risks to avoid social interactions even when they may be safe.

The inclusion of a risk scale to identify where people see risks during the pandemic—whether to the economy, personal or others’ health, freedom, or media integrity—could explain whether people’s fears impact either their conspiracy theory belief and/or their lockdown behaviour. With respect to our study, specific lockdown behaviours include actions that are either compliant or non-compliant with government guidelines, as well as intentions for future preventive behaviours. Compliant behaviours include avoiding large public gatherings and wearing face masks in public. Preventive behaviours include intentions to partake in contact-tracing schemes and getting vaccinated.

By employing a correlational design, this research aims to shed light on the relationship between conspiracy belief and different domains of COVID-19 risk perception; namely, risks around personal health, others’ health, the economy, individual freedom and media integrity. Specifically, we test a parallel mediation model with conspiracy belief as the predictor variable, compliant behaviour as the dependent variable, and the different types of risks as mediators. Through a series of questions asking participants to indicate their levels of perceived risk for these variables, this research can highlight what risk perceptions promote or hinder compliant behaviour, and whether these risk perceptions mediate the impact of conspiracy theory belief on compliance.

To the best of our knowledge, this is the first study testing how belief in conspiracy theories correlates with lockdown behaviour in the UK through its impact on risk perceptions. Participants were specifically pooled from the UK in order to avoid confounds amongst the variability in government regulations across different countries towards the pandemic. Our study tested the following hypotheses:

***Hypothesis 1*:** Belief in conspiracy theories will be negatively associated with compliant behaviours, so that the more people endorse conspiracy theories, the less compliant they will be with governmental guidelines.

***Hypothesis 2*:** Belief in conspiracy theories will be negatively associated with health-related risks and other-centered risks, and positively associated with economic uncertainties, media scepticism, and freedom-suppression related risks.

***Hypothesis 3*:** Risk perceptions will mediate the link between belief in conspiracy theories and compliant behaviours.

## Methods

### Participants and design

Participants were recruited using a snowballing technique primarily on social media between the period of the first and second national lockdown in the UK (24^th^ August– 21^st^ September 2020). Out of the 475 respondents who accessed the survey, 11 were removed because they stated they did not reside in the UK during any point of the COVID-19 lockdown, and 24 respondents were removed from the final analysis for failing the attention check embedded within the survey. A further 72 participants did not fully complete the survey, leaving 368 participants in the final sample.

The age of the sample ranged from 18 to 84 year olds (*M* = 35.05, *SD* = 14.45) and was heavily skewed towards the younger side–modal age was 22 with a significant drop in ages past 30. The sample was 61.7% female. Males accounted for 35.6%, with 1.9% listing gender as ‘other’ and 0.8% not disclosing it. The sample was largely White British, this ethnicity making up 72.79% of the sample. We also asked our participants whether they were employed in the healthcare sector, and whether they or one of their loved ones had contracted COVID-19 up to the point of the study, as we reasoned that these were factors which could potentially affect their responses. We provide more details on these factors in the Results section.

Our study used a correlational cross-sectional online design. Our key outcome variable was a composite of self-reported compliant behaviours during lockdown, with belief in conspiracy theories (both general and COVID-19 specific) being the key predictor. As our mediators for this hypothesised relationship, we measured perceptions about risks to personal health, health of others, media agendas, personal freedom, and economy. We also measured self-reported future preventive behaviours to explore whether current compliance can predict compliance and safe behaviour in the future summarised in exploratory analyses.

### Materials

#### Belief in conspiracy theories scale

The established eight-item Belief in Conspiracy Theories Scale (*e*.*g*., “The attack on the Twin Towers was not a terrorist action but a governmental conspiracy”) was used to measure general belief in conspiracy theories [59]. This scale has been used widely in academic research [60–67]. Previous work has demonstrated its factorial and convergent validity [68], while it has also been used to validate related conspiracy inventories as a basis for criterion validity [69].

We added four conspiracy theory questions which specifically related to coronavirus (*“COVID-19 restrictions are in place for political reasons*,*” “The COVID-19 virus was created in a laboratory*,*” “Masks cause the spread of COVID-19 not prevent it”*, *and “There is a link between COVID-19 and the installation of 5G”*). Responses to these questions were measured on a seven point scale ranging from *1 = strongly disagree* to *7 = strongly agree*. The Belief in Conspiracy Theories Scale and these four additional questions were merged into an adapted Belief in Conspiracy Theory Scale (*α* = .86; the high Cronbach’s alpha value suggested that the adapted scale showed high internal consistency, and the use of the four additional COVID-19 items was therefore deemed acceptable).

#### COVID-19 risk perceptions scale

This scale was developed for the purposes of this study, and asked participants questions about their views on the dangers present during the pandemic and what they deemed to be the most significant risks. Twenty-eight items were included asking people’s degree of agreement with questions relating to health (5 items, *e*.*g*. *“coronavirus is highly deadly*”) (*α* = .83), media integrity (6 items, *e*.*g*. *“the media inflates how dangerous coronavirus really is”*) (*α* = .83), economic concerns (5 items, *e*.*g*. *“more people will die from the poverty that the lockdown will cause than from coronavirus itself”*) (*α* = .85), freedom (7 items, *e*.*g*. *“demanding that people wear masks in public is totalitarian*”) (*α* = .78), and other-centered risks (5 items, *e*.*g*., *“I would feel guilty if I gave someone coronavirus by breaking social distancing”*) (*α* = .81). A seven point scale was used to record participants’ agreement with the statements about these risks (*1 = strongly disagree*, *7 = strongly agree*).

#### Factor determination and item reduction for risk scale

We conducted a Principal Components Analysis (PCA) to test the factor structure of our risk perceptions scale, which was originally designed to measure five distinct factors (health risks, other-centered risks, economic risks, media skepticism, freedom risks). We used a Monte Carlo parallel analysis to determine the number of factors, which has been found to perform better than more traditional methods such as the Kaiser Criterion or scree plots [70]. With 28 items, 368 participants, and 1,000 bootstrapped simulations, the parallel analysis converged on a 3-factor solution.

We therefore conducted a PCA using a Varimax rotation and restricting the number of extracted factors to 3. Both tests of sampling adequacy (KMO = .932) and sphericity (χ^2^ (378) = 5042.25, *p* < .001) were satisfactory, however a determinant value of 5.97^−7^ (< .00001) indicated potential problems with multicollinearity. Upon closer inspection of the inter-item correlation matrix, no inter-correlations of over *r* = .9 were found, indicating that the multicollinearity issues potentially arose from an excessive number of items, and suggesting that item reduction had to be undertaken.

We observed the rotated component matrix and suppressed factor loadings below 0.4. Through a cursory observation, all of the health and other-centered risks loaded quite clearly on a single factor. All economic risks loaded onto a second factor, however some additional items from the original media and freedom sub-scales also loaded on this factor. These items seemed to be centered around liberty, for example *“Demanding that people wear masks in public is totalitarian”* or *“My personal freedom is under threat because of quarantine”*. The third factor consisted mostly of media items, with an additional item loading on this concerning potential secret agendas on the part of governments. Thus, this factor seemed to concern skepticism about the information people received.

Based on the low determinant in the first instance, we removed any items which did not load on any of the 3 factors, or items which cross-loaded between factors. The item *“Socializing online is much worse than socializing in person”* was removed, as it did not load on any factors. Further, the items *“There have been other similar viruses in the past*, *but none of them received media attention”*, *“The media inflates how dangerous Coronavirus really is”*, and *“Quarantine is too extreme a measure”* loaded on two factors and were also removed. Upon closer inspection of our scales, we also noted that two of the items in the original other-centered scale, namely *“I would urge people close to me to stay at home”* and *“I would NOT visit an elderly family member during the pandemic”* concerned behaviours, and not necessarily risk perceptions. As such, these two items were also removed prior to re-running the PCA.

#### Exploratory PCA and determination of risk factors

With the reduced 23-item scale, we re-ran the PCA restricted to three factors (optimal factor solution was still 3 with 23 items). Once again, sampling adequacy (KMO = .908) and Bartlett’s sphericity test (χ^2^ (253) = 3720.34, *p* < .001) were satisfactory. However, this time we also observed a satisfactory determinant (= .00003), indicating that multicollinearity issues were successfully addressed through our scale reduction. Once again, we restricted factor loadings to a minimum of 0.4, and the factor solution to 3 factors, following the parallel analysis. We identified three key risk perceptions through this analysis: 1) coronavirus health concerns, which explained 34.93% of the variance, 2) economy and liberty risks, which explained 9.05% of the variance, and 3) informational risks, which explained 6.80% of the variance. Respectively, these pertained to perceived risks about one’s own health and the health of others, risks around adverse economic effects and impacts on human liberties, and risks with respect to media and government transparency in the information provided to the public with respect to COVID-19. The items constituting these components are shown in Table 1, along with their Cronbach’s alpha values.

**Table 1:** Risk perception sub-scales with final factor solutions.

Factor	Item	Factor loading
Coronavirus health risks (α = .871)	The prospect of contracting coronavirus scares me.	.739
	Coronavirus is highly deadly.	.630
	Even if coronavirus does not kill you, it might cause long-term health problems.	.736
	I would worry about my life if I contracted Coronavirus.	.705
	Coronavirus is serious enough to send you to the hospital.	.621
	I am afraid someone close to me might contract Coronavirus.	.742
	I would feel guilty if I gave someone Coronavirus by breaking social distancing.	.628
	If someone close to me got Coronavirus, I would be worried about them.	.733
Economy and liberty risks (α = .864)	Having a salary is more important than protecting yourself from Coronavirus.	.645
	More people will die from the poverty that lockdown will cause than from Coronavirus itself.	.593
	The economy should be a more primary concern than Coronavirus.	.764
	Saving the economy is more important than eradicating Coronavirus.	.764
	Worldwide economic collapse will create more problems than a global pandemic.	.766
	Quarantine will end society as we know it.	.473
	My personal freedom is under threat because of quarantine.	.591
	Censoring opinions which go against government guidelines is a violation of human rights.	.452
	Demanding that people wear masks in public is totalitarian.	.508
Informational risks (α = .729)	The media is doing a bad job of informing the public about Coronavirus risks.	.750
	Without the media, we would have fewer Coronavirus deaths.	.670
	The media cannot help in controlling the spread of the virus.	.567
	News channels are only pushing agendas when it comes to Coronavirus.	.667
	I fear that the government is using Coronavirus to serve other agendas.	.574

#### Compliant behaviours scale

The compliant behaviours scale, which we developed for this study, asked participants about the regularity with which they performed behaviours either directly forbidden by the government or recommended by the government as measures that would reduce the risk of infection and spreading the virus (*e*.*g*., *“Please estimate how often you wore a face mask in public”*). The scale originally consisted of 13 items, however, one of the items which concerned protesting about 5G towers was found to display floor effects and was removed. This left 12 items, measured on seven-point scales ranging from *1 = never* to *7 = always* (*α* = .81).

#### Future preventive behaviours scale

The scale was designed to gauge the likelihood of participants engaging in behaviours that would reduce their chance of infection or transmission. These included questions related to track and trace schemes, willingness to be vaccinated, and whether they would get tested (*e*.*g*., *“To what extent do you agree that you would take part in a contact tracing scheme*?*”*). The original scale consisted of 6 items, however one of the items which concerned protecting against the virus with a meat-free diet was found to have little internal validity following participant feedback and was removed, leaving five items rated on seven-point scales ranging from *1 = strongly disagree* to *7 = strongly agree* (*α* = .75).

#### Political orientation scale

The scale consisted of one item which asked participants to place themselves on a political orientation spectrum using a slider scale, from -1 for extremely liberal to 100 for extremely conservative. Full scales can be found in the S1 Appendix.

### Procedure

Participants were asked to fill out a consent form expressing their willingness to take part and have their data stored anonymously. Participants first provided their demographics: age, gender and ethnicity. Participants also specified whether they had been living in the UK during any point of the lockdown so as to avoid any confounds of the varying approaches different countries and governments took to combat COVID-19. Answering “no’’ to this question terminated the survey, and these responses were discarded from the final analysis. The final questions in the demographic section of the survey asked participants about their political orientation. In the main body of the survey, participants were presented with the compliant behaviours scale, followed by the future preventive behaviours scale. As potential covariates to address, questions were asked at this point about whether participants had contracted the virus, knew someone who contracted the virus, or worked in a medical field. In the final two sections of the main body of the survey, participants were asked about their risk perceptions in relation to COVID-19 and presented with an adapted version of the Belief In Conspiracy Theories Scale which included additional questions about belief in COVID-19 conspiracies developed for this study. Scales were presented in this order, with the behaviours scales first, followed by the risk perception scale and finally the Belief in Conspiracy Theories Scale to avoid the consideration of risks or conspiracy theories biasing participants’ answers. Upon completion of the survey, participants were fully debriefed.

## Results

### Analytical plan

After reducing our risk scale using the PCA reported above, we conducted simple one-way ANOVAs to observe whether other factors, such as respondents’ professions or previous experiences with COVID-19, could have affected their responses. For our inferential testing, we examined the ability of all our predictors (belief in conspiracy theories and all risk factors) to predict compliant behaviours. Finally, we examined the role of all risk factors as potential mediators of the association between belief in conspiracy theories and compliant behaviours. The analysis plan was pre-registered prior to data collection: https://aspredicted.org/RHQ_YTB.

### Descriptive statistics

We provide descriptive statistics for each of the scale variables, as well as the correlations between the items in Table 2. To explore potential relationships, we also present correlations and descriptive statistics for the age and political orientation demographic variables.

**Table 2:** Correlation matrix.

	1	2	3	4	5	6	7	8	*M*	*SD*
1. Age	-	-.087	.026	.000	-.052	-.013	-.022	-.155**	35.05	14.45
2. Politics		-	-.269**	.319**	.185**	-.094	-.217**	.284**	28.80	21.43
3. Health risks			-	-.546**	-.393**	.558**	.583**	-.301**	5.32	1.08
4. Economic & liberty risks				-	.602**	-.398**	-.456**	.439**	3.68	1.07
5. Informational risks					-	-.263**	-.458**	.521**	3.43	1.13
6. Compliant behaviours						-	.471**	-.136**	5.39	.84
7. Preventive behaviours							-	-.484**	6.07	.96
8. Conspiracy belief								-	2.50	.91

*Note*.

**p < .01 (two-tailed). For politics scale, -1 = fully liberal, 100 = fully conservative. All scale variables’ potential values range from 1 to 7.

### Exploratory analyses for sample effects

Given the finalization of the risk factors following the factor analysis, we tested whether some sample characteristics could have affected responses to these; namely, whether participants were employed in a medical field or not, and whether they, or someone they knew, had contracted coronavirus up to the time of them taking the survey.

By profession, 21 out of our 368 participants reported working in a medical field. Because of the large imbalance between the two populations, we do not report any profession-based analyses here as results are unlikely to be meaningful.

In our sample, 13 participants reported having contracted COVID-19, with 111 reporting they had not, and a further 244 saying they were not sure. We conducted one-way ANOVAs to test whether there were any significant differences between these groups regarding risk perceptions. None of these detected any significant differences (health *p* = .868; economy and liberty *p* = .188; informational risk *p* = .910). Furthermore, 89 participants reported having family members or close people who contracted COVID-19, while 83 said they did not and 196 said they were not sure. Similarly, a series of one-way ANOVAs examining this variable found no significant effects on risk perceptions (health *p* = .099; economy and liberty *p* = .107; informational risk *p* = .353).

For robustness, we report the same analyses with non-parametric equivalents (Kruskal-Wallis tests) in S2 Appendix. The results of these analyses are consistent with the ANOVA results reported above.

### Inferential tests

#### Multiple regression model

We conducted multiple regression to test whether all predictors (health risks, economic and liberty risks, informational risks, belief in conspiracy theories) were associated with compliant behaviours. All predictors were entered into a single step. This model was significant, *F* (4, 354) = 44.21, *p* < .001, with the predictors explaining 33.6% of the variance in compliant behaviours (adjusted *R*^*2*^ = .336). However, as can be seen from Table 3, when accounting for the variance of all other predictors, only health risks (positive) and economic and liberty risks (negative) were significantly associated with compliant behaviours. In other words, those who perceived lower health and higher economic and liberty risks were less likely to engage with compliant behaviours. The effects of belief in conspiracy theories and informational risks on compliant behaviours were not statistically significant.

**Table 3:** Regression coefficients for compliant behaviours.

Predictor variable	B	SE	*β*	*t*	*p*
Health risks	.391	.041	.502	9.60	< .001
Economic & liberty risks	-.124	.048	-.157	-2.56	.011
Informational risks	-.006	.044	-.009	-.146	.884
Belief in conspiracy theories	.084	.048	.090	1.73	.084

#### Mediation model

To test the hypothesis that belief in conspiracy theories is associated with compliant behaviours through its effect on risk perceptions, we tested a parallel mediation model using Hayes’ PROCESS macro (Model 4) [71]. Belief in conspiracy theories was tested as the distal predictor, with the three risk factors (health, economic & liberty, informational) as parallel mediators and compliant behaviours as the outcome variable. Using 5,000 bootstrapped samples at a 95% confidence interval (Davidson-MacKinnon heteroscedasticity consistence), this model was significant and explained 33.6% of the variance in compliant behaviours, *F* (4, 350) = 32.95, *p* < .001, *R*^2^ = .336.

**Fig 1:**
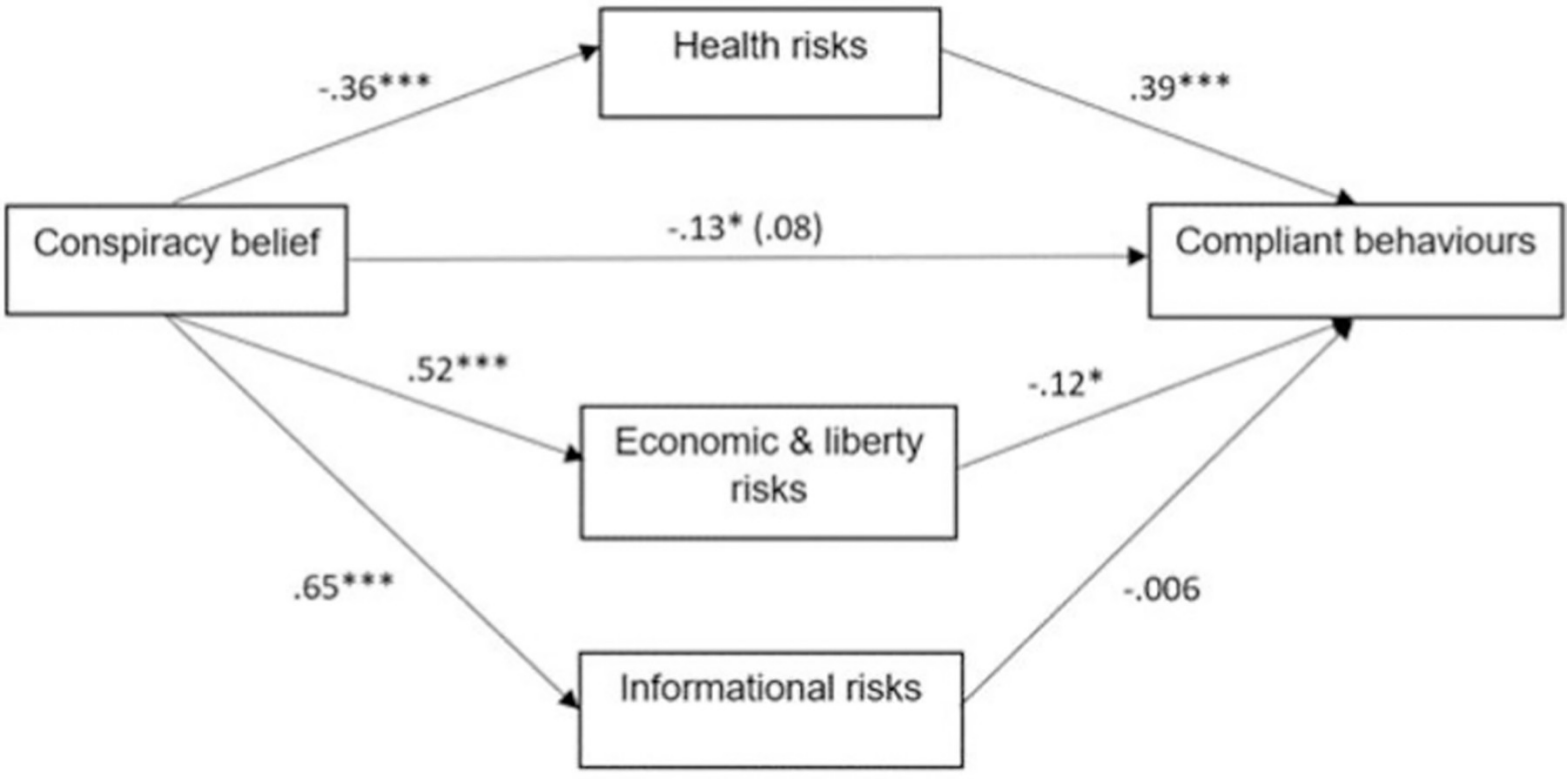
Parallel mediation model with conspiracy belief as the distal predictor. *p < .05. ***p < .001.

The significant total effect of belief in conspiracy theories on compliant behaviours (*B* = -.13, *SE* = .06, *t* = -2.08, *p* = .039, 95% CI [-.24, -.01]) became non-significant in the direct model (*B* = .08, *SE* = .05, *t* = 1.67, *p* = .095, 95% CI [-.01, .18]). The direct paths of health risks (*B* = -.39, *SE* = .05, *t* = 8.32, *p* < .001, 95% CI [.30, .48]) and economic & liberty risks (*B* = -.12, *SE* = .05, *t* = -2.41, *p* = .02, 95% CI [-.22, -.02]) on compliant behaviours were statistically significant, whereas the effect of informational risks was not (*B* = -.006, *SE* = .04, *t* = -.15, *p* = .88, 95% CI [-.09, .08]; see Table 4). Similarly, the indirect effects were only statistically significant for health (*B* = -.14, *SE* = .04, 95% CI [-.21, -.07]) and economic risks (*B* = -.06, *SE* = .03, 95% CI [-.12, -.02]), whilst the indirect effect was not statistically significant for informational risks (*B* = -.004, *SE* = .03, 95% CI [-.06, .05]). That is, health risks and economic and liberty risks (but not informational risks) significantly mediated the association between belief in conspiracy theories and compliant behaviours. The total indirect effect of the model was also statistically significant, *B* = -.22, *SE* = .05, 95% CI: [-.32, -.13]. In other words, the more participants believed in conspiracy theories, the less they perceived health risks and the more they perceived economic and liberty risks, and in turn, the less likely they were to engage with compliant behaviours (Fig 1 and Table 4). As a sensitivity analysis, we repeated the above mediation while including age as a covariate (S3 Appendix). This did not change the general pattern or magnitude of effects reported here.

**Table 4:** Path coefficients.

Outcome variable	Path variable	*B*	*SE*	*t*	*p*	95% CI
Health risks	Belief in conspiracy theories	-.36	.08	-4.66	< .001	[-.51, -.21]
Economy & liberty risks	Belief in conspiracy theories	.52	.06	8.51	< .001	[.40, .64]
Informational risks	Belief in conspiracy theories	.65	.06	11.30	< .001	[.54, .77]
Compliant behaviour	Belief in conspiracy theories	.08	.05	1.67	.10	[-.01, .18]
	Health risks	.39	.05	8.32	< .001	[.30, .48]
	Econ. & liberty risks	-.12	.05	-2.41	.02	[-.22, -.02]
	Informational risks	-.006	.04	-.15	.88	[-.09, .08]
Compliant behaviour (total effect)	Belief in conspiracy theories	-.13	.06	-2.08	.04	[-.24, -.007]

*Exploratory analysis of behaviour-intention consistency*. As a further step, we also tested for the ability of current compliant behaviours to predict self-reported likelihood of future preventive behaviours. This variable did not form part of our main analysis, hence it was not included in the mediation model. Rather, we explored the potential for current behaviours relating to COVID-19 to predict how people believe they will act in the future as a proxy for consistency of pandemic-related behavioural intentions over time. In turn, future studies can examine if the opposite is true (*i*.*e*., whether current behaviours are predicted by retrospective behaviours) to verify this.

This simple regression model was statistically significant, *F* (1, 362) = 102.66, *p* < .001, and compliant behaviours explained 22.1% of the variance (R^2^ = .221) in future preventive behaviours. Compliant behaviour was a significant predictor of future preventive behaviours (*B* = .539, *SE* = .05, *t* = 10.13, *p* < .001), with a coefficient *B* = 3.16 (*SE* = .29, *t* = 10.88, *p* < .001). However, we must note that the future preventive behaviours scale showed an extreme negative skew, indicating the existence of ceiling effects. We therefore advise caution in interpreting this analysis. Many of the actions regarded as future possibilities in this scale (*e*.*g*., vaccination, track-and-trace app downloads) have since been rolled out, therefore research is currently at a position where this predictive relationship could also be verified retrospectively with administrative data arising from vaccination and track-and-trace drives.

## Discussion

In this study, we investigated whether belief in conspiracy theories and risk perceptions were associated with compliance to pandemic-related guidelines. The mediation model tested largely supported our hypotheses. In support of Hypothesis 1, we found a significant association between conspiracy beliefs and compliant behaviours, so that the more participants believed conspiracy theories the less frequently they engaged with compliant behaviours. However, when controlling for risk perceptions, the association between belief in conspiracy theories and compliant behaviour was no longer statistically significant. Hypothesis 2 was also supported since all of the resulting risk factors were related to conspiracy beliefs, in directions consistent with what was hypothesised regarding their constituent factors. Specifically, conspiracy beliefs were negatively related to health risks (consisting of both personal and others’ health), and positively related to economic and liberty risks, as well as informational risks. Furthermore, our results largely support Hypothesis 3 as health risks and economy and liberty risks were significant mediators of the relationship between conspiracy belief and compliant behaviour. However, informational risks were found not to be a statistically significant mediator of this relationship.

The present research helps to understand why previous studies on the association between risk perceptions and behaviour during the COVID-19 lockdown have not been unanimous up to this point [51–56]. Whilst health related risks positively correlated with compliant behaviour, other risk perceptions such as economic and liberty risks negatively correlated with the likelihood of following government guidelines. Although previous literature has taken risk perceptions as a single measure, this research broke down risk perceptions into different categories and identified the importance not only of gauging the perception of risk but also clarifying what the actual perceived risks are, and how they individually correlate with behaviour.

Whilst previous research found that different COVID-19 related conspiracy theory beliefs may lead to different behavioural outcomes [8, 31, 32], our research extends prior studies by elucidating risk perceptions as one of the mechanisms that drive the association between belief in conspiracy theories and compliant behaviour. This demonstrates that belief in conspiracy theories may act through certain mechanisms to bring about behaviour; namely, in how belief in conspiracies correlates with subjective perceptions of different types of risks. Such nuances may be important to understand, given that conspiracy beliefs may be deeply ingrained and difficult to change [72].

### Implications

Conspiracy beliefs correlated with different types of risk perceptions in different directions. For example, our findings suggest that conspiracy beliefs are associated with downplaying health risks and amplifying perceived economic, liberty, and informational risks. Because we have also found negative correlations between health risks and the other types of risks we examined here, it is possible that conspiracy beliefs create this apparent mutual exclusivity. Taking conspiracy beliefs as a root construct driving risk perceptions, it is likely that interventions which promote trust and thus reduce the need to seek alternative explanations can also promote more accurate perceptions of different kinds of risk [26, 73]. However, it may also be the case that explaining COVID-19 risks from different angles, for example the need to adhere to measures for a quick economic recovery, can be a worthwhile approach. If different messages can be targeted to groups with varying risk perceptions, governments could therefore promote the same desired behavioural outcome through appealing to different ways of perceiving the risks and allow countries to achieve their goal of a quick recovery from the pandemic. Indeed, recent work has demonstrated that messaging that is tailored to individuals’ risk perceptions can be more effective in driving up vaccination uptake [74].

The negative correlation between health, and liberty and economic risk perceptions may also support the ‘Finite pool of worry’ hypothesis, which states that individuals only have the capacity for a certain amount of concern before having to prioritise some issues to alleviate the stress of others [75]. Whilst originally applied to climate change anxiety, this phenomenon [76, 77] could extend to other crises that are all encompassing and affect individuals’ abilities to accurately assess the threats and risks that impact daily life. Indeed, a recent study has highlighted that lower-income individuals are more likely to hold misperceptions about the risk that COVID-19 poses to themselves [78]. Whilst other factors such as educational levels could be confounding this relationship, it is also possible that lower-income individuals prioritise economy-related risks over health-related ones. Especially since conspiracy belief was related to all of the risk perceptions we considered in different directions, it may be the case that such perceptions are generated from conspiracy beliefs in order to cope with the crisis and justify more generalized belief systems since most of these risk perceptions acted as mediating mechanisms in the relationship between conspiracy beliefs and compliant behaviours.

Furthermore, our research adds some nuance to the study of conspiracy beliefs as drivers of pandemic-related behaviour. Given the finding that risk perceptions mediate the association between belief in conspiracy theories and compliance behaviour, it is likely that differing levels of conspiracy beliefs give rise to polarisation of not only opinions about the pandemic, but also of risk perceptions themselves [79]. To this end, popularising different risk perceptions by different groups of people may sensitise the general public to other opinions about COVID-19. For example, non-compliant people may be sensitised if they are exposed to the health risks perceived by a person in high-risk groups, whereas hard lockdown advocates may be sensitised through exposure to people who have lost their livelihoods throughout the pandemic, and thus adopt higher economic risk perceptions. By identifying risk perceptions as mediating mechanisms, we introduce more vectors which can be targeted to ease COVID-related conversations and drive higher trust and compliance.

### Limitations and future research

This research has identified a potential gap in the literature around the impact of belief in conspiracy theories and compliant behaviour through the introduction of risk perceptions as mediators. By testing a variety of risk perceptions, this research has the benefit of drawing more nuanced conclusions about how beliefs in conspiracy theories may be related to compliant behaviour. Additionally, our study uniquely evaluates a sector of the UK population at a crucial lockdown period. Since our data were collected during a UK lockdown period, the results reflect responses from that period of time that would now be difficult to obtain.

However, this research is also subject to some limitations. One could reason that our risk scales, particularly the media and economy-related scales, were framed in a way that made them mutually exclusive to the health or other-centered scales (*e*.*g*., *“the economy should be a more primary concern than coronavirus”*) and thus could have influenced responses. However, it is important to note that none of the questions on media or the economy concerned the actual health risk of COVID-19. Instead, these questions merely measured the extent to which people thought the burdens that lockdown would impose on the economy made sense given the nature of the pandemic. Our findings suggest that overall, participants may have viewed health risks associated with coronavirus as mutually exclusive with the economic and liberty risks brought about by lockdown. Nonetheless, while we attempted to validate the factor structure of our risk perception scale, we recognise that we were unable to fully demonstrate our scale’s external validity. With respect to Table 1, we observed that the risk factors correlated with other constructs and self-reported behaviours in expected directions, however future research may wish to replicate this using alternative measurements of risk perceptions.

Furthermore, it is possible that belief in conspiracy theories may vary according to the information sources that people seek or are exposed to. Social media contributes to the spread of conspiracy theories [22] given the contribution to echo chambers [80, 81]. Likewise, it is possible that different social categories such as socioeconomic status and education may influence the degree to which people endorse conspiracy theories and have differential effects on different risk perceptions. However, given that we did not collect this information, we were unable to account for their potential effects on the tested model. Both are fruitful areas for future exploration in follow up research.

In this study we found support for our hypothesized model. We hypothesized the direction of the effects based on evidence that beliefs in conspiracy theories explain risk perceptions [9, 19, 20, 72, 80–83] and that in turn, risk perceptions are predictors of behaviour [84–86]. However, given the correlational nature of the data, we cannot speak for causality and further research employing experimental and longitudinal designs would be important to establish causality and provide further support for the direction of the effects. Taking the different links individually, we can start to predict their direction. The connection identified between belief in conspiracy theories and behaviour can feasibly be predicted due to the prevalence of research that investigates the influence of conspiracy beliefs on behaviour. Experimental research in which conspiracy theory belief was manipulated has found it reduces participants’ intentions to engage in political and sustainable behaviours [20], and influences mediators of unethical behaviour [80, 81]. Furthermore, anti-vaccination conspiracy theories facilitate vaccine hesitancy [82–84] especially during the COVID-19 pandemic [85], and negatively impact vaccination intentions [9, 19, 72, 82, 83]. Research into the effect beliefs and risk perceptions have on behaviour within health also allow us to reasonably predict that risk perceptions [86] and perceptions of severity of risk can predict behaviour [85, 86]. Future experimental research is needed to investigate whether belief in conspiracy theories is predictive of COVID-19 related compliant behaviours; however, based on previous research, we can reasonably hypothesise that the direction of the association between these variables is the one tested in the present study.

Participants were younger and more liberal, most likely as a consequence of the non-randomised nature of snowball sampling. This is an important consideration, since both the risk perceptions we were measuring and belief in conspiracy theories were closely related to political orientation. Our pattern of results may therefore be more applicable to those segments of the population who are left-leaning.

Future research could benefit from a more representative sample. Demographic characteristics may impact pandemic behaviours, as differences have been found in previous outbreaks. During the early stages of the avian influenza outbreak, younger people were more willing to travel and continue going on vacation as opposed to older people [42], and another study from the H1N1 pandemic reported that respondents from larger households took part in more precautionary behaviours [87]. Therefore, general demographics could influence risk perceptions and resulting behaviours, and future research to predict increased preventive behaviour compliance could identify the influence that these factors play more holistically. Previous research has demonstrated that effective messaging often has to be tailored to its audience, and a one size fits all messaging strategy will not be universally effective, indicating that different groups may have different priorities and therefore different risk perceptions [88].

Future studies could survey risk perceptions of who accepted and refused vaccination as an opportunity to test our model’s predictive ability retrospectively. This research could consist of surveying people who have and have not attended their vaccine invitation on their conspiracy beliefs, risk perceptions, and current compliance with offered immunisation.

Furthermore, it remains unclear whether the risk perceptions we observed here were due to underlying motivations. Conflicts between the perceived health risk of the pandemic itself, for example, and the risk of a faltering economy which can jeopardise one’s livelihood, may introduce a state of internal turmoil, also known as cognitive dissonance. Cognitive Dissonance Theory claims psychological discomfort occurs when an individual’s thoughts or attitudes are inconsistent, especially when this leads to behavioural changes [89, 90]. In order to relieve this state of tension, individuals work to change one or both of their cognitions to be consistent again [91, 92]. Therefore, someone experiencing this pandemic-related cognitive predicament might rationalize their behaviour of not wearing a mask by distorting their cognition about masks protecting others’ health and finding research that claims masks do not aid in disease prevention. These rationalizations can be problematic as opposing sides continue to find information to support their own side, which may further polarise media platforms and subsequently public opinion. Since cognitive dissonance was not expressly tested in this study, future research could examine this speculation.

Finally, this study was conducted between the first and second national UK lockdowns, which may have been a very unique period in terms of available information, protective measures in place, and the degree to which belief systems about COVID-19 had been fully established. While our results largely echo anecdotal observations at the time, namely less compliant people making reference to reopening the economy and regaining their freedoms, it is likely that such risk perceptions have shifted with newer developments such as the reopening of entertainment venues and the rollout of vaccination certificates. Future studies which adopt a longitudinal approach across multiple countries which are at different stages in the pandemic can be uniquely equipped to verify whether this is the case, by tracking risk perceptions over time and in relation to changing measures. This would constitute valuable evidence in determining whether risk perceptions are also a function of people’s need to justify their stance against (or in support of) COVID-19 measures.

## Conclusion

This study adds to our understanding of how beliefs in conspiracy theories and differing perceptions of risk are associated with people’s behaviour during a health pandemic. Our findings reconcile the mixed results reported thus far about the impact of risk perceptions on behavioural outcomes during the covid pandemic. Individuals who considered public and personal health as the most serious and pervasive risk were much more willing to comply with the UK government’s recommended behaviours. Whilst belief in conspiracy theories negatively correlated with compliant behaviour replicating prior research, we also found that this effect is mediated by individual risk perceptions. Whereby health related risk perceptions were positively correlated with compliant behaviour, belief in conspiracy theories was negatively associated with risk perceptions of health, positively associated with economic and personal liberty risk perception, and associated with lower pandemic-related government compliance. These findings can be used to develop more effective and better targeted messaging and interventions to promote future compliance with government measures taking into account that coronavirus is in fact both a significant risk to people’s health and to the nation’s economy.

## Supporting information

S1 Appendix Qualtrics survey. (PDF)

S2 Appendix Kruskal-Wallis sensitivity analysis. (DOCX)

S3 Appendix Parallel mediation sensitivity analysis with age as covariates. (DOCX)

S1 Data Fully anonymised dataset. (CSV)
