# Supplementary material for: The impact of risk perceptions and belief in conspiracy theories on COVID-19 pandemic-related behaviours
Source: PLoS One. 2022 Feb 8;17(2):e0263716. doi: 10.1371/journal.pone.0263716 (PMC8824369; doi:10.1371/journal.pone.0263716)
Supplement: S1 Appendix — (PDF) [file pone.0263716.s001.pdf]

Participant Information Sheet

Participant Information Sheet
Project title: Experiences during the COVID-19 Pandemic

Researcher(s): Sara Komer, Alexandros Efstratiou, Jack Hughes, Lilli Baxter
Contact details: sara.komer@durham.ac.uk, alexandros.efstratiou@durham.ac.uk, jack.p.hughes@durham.ac.uk, lilli.a.baxter@durham.ac.uk

Supervisors name: Dr Ana Leite and Dr. Milica Vasiljevic
Supervisors contact details: Ana.Castro-Leite@durham.ac.uk, Milica.Vasiljevic@durham.ac.uk

This study has received ethical approval from the ethics committee of Durham University.

Before you decide whether to agree to take part it is important for you to understand the purpose of the research and what is involved as a participant. Please read the following information carefully. Please get in contact via the email addresses provided above if there is anything that is not clear or if you would like more information.

What is the purpose of the study?
The aim of this study is to investigate people's experiences during the COVID-19 pandemic. It should take around 10 minutes to complete.

Why have I been invited to take part?
You have been invited because you are currently residing in the UK.

Do I have to take part?
Your participation is voluntary and you do not have to take part. You can withdraw at any time by closing the browser window without giving a reason. If you close the browser window before you finish the survey none of your responses will be used in the analyses. If you wish to withdraw your responses from this study after completion please email the

researcher within one week of completion, quoting your unique participation code (created for the purpose of this study). Please note that if you contact us after that deadline we will not be able to delete your responses. We will not record any details regarding your IP address.

What will happen to me if I take part?
If you agree to take part in the study, you will answer the questionnaire provided. You can omit any questions you do not wish to answer at any time during the survey.

Are there any potential risks involved?
We don't anticipate any risks arising from participating in this study, other than those encountered in normal life.

Will my data be kept confidential?
The data you provide is fully anonymous and we will not collect or ask you to provide any personal data. We will have no way of linking responses back to an individual. We will not connect data to the IP address from which the survey was completed.

What will happen to the results of the project?
All responses are anonymous and will only be reported in an aggregate format (by reporting only combined results and never reporting individual ones). Anonymised data may be used in publications, reports, presentations and other research outputs. Data will be treated confidentially and any publication resulting from this work will report only data that does not identify individual participants. Participants' anonymised responses, however, may be shared with other researchers or made available in online data repositories.
All research data and records needed to validate the research findings will be stored for 10 years after the end of the project.

Who do I contact if I have any questions or concerns about this study?
If you have any further questions or concerns about this study, please email the researchers or their supervisors via the contact details provided above.

Thank you for reading this information and considering taking part in this study.

Consent

If you would like to take part please complete the form below to confirm that you understand what the purposes of the project are, what is involved and that you are happy to take part.

- [ ] I confirm that I have read and understand the information sheet for this project.
- [ ] I have had sufficient time to consider the information and ask any questions I might have, and I am satisfied with the answers I have been given.
- [ ] I understand who will have access to data provided, how the data will be stored and what will happen to the data at the end of the project.
- [ ] I understand that my participation is voluntary and that I am free to withdraw at any time without giving a reason.
- [ ] I understand that my anonymised data may be archived and shared with others for legitimate research purposes.
- [ ] I agree to take part in this project.

Anonymous code

Please create a unique anonymous code and note it down yourself so that should you wish to withdraw your data after completing the questionnaire you can supply this to the researcher.

This can be the last two letters of your surname, your year of birth and first two letters of your mother or fathers name (for example John Smith 1995 mother Jane Smith: TH1995JA)

Demographics (Base/Universal)

How would you describe your gender?

- ○ Male
- ○ Female
- ○ Other
- ○ Prefer not to say

How old are you?

What is your ethnic group?

White

- English/Welsh/Scottish/Northern Irish/British
- Irish
- Gypsy or Irish Traveller
- American

Mixed/ Multiple Ethnic Groups

- White and Black Carribean
- White and Black African
- White and Asian
- Any other mixed/multiple ethnic group background

Asian/ Asian British

- Indian
- Bangladeshi
- Pakistani
- Chinese
- Any other Asian background

Black/ African/ Caribbean/ Black British

- African
- Carribean
- Black British
- African American
- Any other Black/African/Caribbean background

Other ethnic group

- Native Hawaiian or Pacific Islander
- Native American or Alaska Native
- Arab
- Any other ethnic group
- Prefer not to say

Were you resident in the UK during any point of the COVID-19 lockdown?

- Yes

○ No

Demographics (Political)

Did you vote in the last election?

○ Yes
○ No

Here is a scale on which the political views that people might hold are arranged from extremely liberal (left) to extremely conservative (right). Where would you place yourself on this scale?

	Liberal (left)	Conservative (right)	
Political Ideology			

Dangerous behaviour matrix

The following questions refer to the COVID-19 pandemic. Please estimate how regularly you performed these behaviours during lockdown:

	Never	Rarely	Sometimes	About half the time	Often	Most of the time	Always
Wore a face mask in public	○	○	○	○	○	○	○
Maintained social distance from other people	○	○	○	○	○	○	○
Stayed at home as much as possible	○	○	○	○	○	○	○

	Never	Rarely	Sometimes	About half the time	Often	Most of the time	Always
Visited friends	○	○	○	○	○	○	○
Protested 5G towers	○	○	○	○	○	○	○

The following questions refer to the COVID-19 pandemic. Please estimate how regularly you performed these behaviours during lockdown:

	Never	Rarely	Sometimes	About half the time	Often	Most of the time	Always
Washed my hands	○	○	○	○	○	○	○
Used hand sanitiser in public	○	○	○	○	○	○	○
Shook hands	○	○	○	○	○	○	○
Touched my face in public	○	○	○	○	○	○	○
Refrained from touching things in shops	○	○	○	○	○	○	○

The following questions refer to the COVID-19 pandemic. Please estimate how regularly you performed these behaviours during lockdown:

	Never	Rarely	Sometimes	About half the time	Often	Most of the time	Always
Sought out hand sanitiser in stores	○	○	○	○	○	○	○
Disinfected my home	○	○	○	○	○	○	○
Avoided public transport	○	○	○	○	○	○	○

Matrix Future preventative behaviours

The following questions are in reference to COVID-19. Please indicate how much you agree with the following statements:

	Strongly disagree	Disagree	Somewhat disagree	Neither agree nor disagree	Somewhat agree	Agree	Strongly agree
I would take part in a contact tracing scheme	○	○	○	○	○	○	○
I would get vaccinated once a vaccine is available	○	○	○	○	○	○	○
I would get tested for Coronavirus	○	○	○	○	○	○	○

The following questions are in reference to COVID-19. Please indicate how much you agree with the following statements:

	Strongly disagree	Disagree	Somewhat disagree	Neither agree nor disagree	Somewhat agree	Agree	Strongly agree
I would self isolate if I suspected I had Coronavirus	○	○	○	○	○	○	○
I would avoid contracting the virus by eating meat free meals	○	○	○	○	○	○	○

	Strongly disagree	Disagree	Somewhat disagree	Neither agree nor disagree	Somewhat agree	Agree	Strongly agree
I would research Coronavirus symptoms	◯	◯	◯	◯	◯	◯	◯

Risks belief control questions

Please answer these questions to the best of your knowledge:

Have you contracted COVID-19?

- ◯ Yes
- ◯ Not sure
- ◯ No

Have any of your family members or close friends/people contracted COVID-19?

- ◯ Yes
- ◯ Not sure
- ◯ No

Do you work in a medical field (e.g. nurse, doctor, biological sample analyst)?

- ◯ Yes
- ◯ No

Covid risk perceptions Matrix

Please specify how much you agree with each of the below statements

	Strongly disagree	Disagree	Somewhat disagree	Neither agree nor disagree	Somewhat agree	Agree	Strongly agree
The prospect of contracting Coronavirus scares me.	○	○	○	○	○	○	○
Coronavirus is highly deadly.	○	○	○	○	○	○	○
Even if coronavirus does not kill you, it might cause long-term health problems.	○	○	○	○	○	○	○
I would worry about my life if I contracted Coronavirus.	○	○	○	○	○	○	○
Coronavirus is serious enough to send you to the hospital.	○	○	○	○	○	○	○

Please specify how much you agree with each of the below statements

	Strongly disagree	Disagree	Somewhat disagree	Neither agree nor disagree	Somewhat agree	Agree	Strongly agree
I am afraid someone close to me might contract Coronavirus.	○	○	○	○	○	○	○

	Strongly disagree	Disagree	Somewhat disagree	Neither agree nor disagree	Somewhat agree	Agree	Strongly agree
I would feel guilty if I gave someone Coronavirus by breaking social distancing.	○	○	○	○	○	○	○
If someone close to me got Coronavirus, I would be worried about them.	○	○	○	○	○	○	○
I would urge people close to me to stay at home.	○	○	○	○	○	○	○
I would NOT visit an elderly family member during the pandemic.	○	○	○	○	○	○	○

Please specify how much you agree with each of the below statements

	Strongly disagree	Disagree	Somewhat disagree	Neither agree nor disagree	Somewhat agree	Agree	Strongly agree
Having a salary is more important than protecting yourself from Coronavirus.	○	○	○	○	○	○	○
This is an attention check. Please select "Somewhat Agree"	○	○	○	○	○	○	○

	Strongly disagree	Disagree	Somewhat disagree	Neither agree nor disagree	Somewhat agree	Agree	Strongly agree
More people will die from the poverty that lockdown will cause than from Coronavirus itself.	○	○	○	○	○	○	○
The economy should be a more primary concern than Coronavirus.	○	○	○	○	○	○	○

Please specify how much you agree with each of the below statements

	Strongly disagree	Disagree	Somewhat disagree	Neither agree nor disagree	Somewhat agree	Agree	Strongly agree
Saving the economy is more important than eradicating Coronavirus.	○	○	○	○	○	○	○
Worldwide economic collapse will create more problems than a global pandemic.	○	○	○	○	○	○	○
There have been other similar viruses in the past but none of them received media attention.	○	○	○	○	○	○	○

	Strongly disagree	Disagree	Somewhat disagree	Neither agree nor disagree	Somewhat agree	Agree	Strongly agree
The media is doing a bad job of informing the public about Coronavirus risks.	○	○	○	○	○	○	○
Without the media, we would have fewer Coronavirus deaths.	○	○	○	○	○	○	○

Please specify how much you agree with each of the below statements

	Strongly disagree	Disagree	Somewhat disagree	Neither agree nor disagree	Somewhat agree	Agree	Strongly agree
The media cannot help in controlling the spread of the virus.	○	○	○	○	○	○	○
The media inflates how dangerous Coronavirus really is.	○	○	○	○	○	○	○
News channels are only pushing agendas when it comes to Coronavirus.	○	○	○	○	○	○	○
Quarantine will end society as we know it.	○	○	○	○	○	○	○
Quarantine is too extreme a measure.	○	○	○	○	○	○	○

Please specify how much you agree with each of the below statements

	Strongly disagree	Disagree	Somewhat disagree	Neither agree nor disagree	Somewhat agree	Agree	Strongly agree
Socialising online is much worse than socialising in person.	○	○	○	○	○	○	○
My personal freedom is under threat because of quarantine.	○	○	○	○	○	○	○
Censoring opinions which go against government guidelines is a violation of human rights.	○	○	○	○	○	○	○
I fear that the government is using Coronavirus to serve other agendas.	○	○	○	○	○	○	○
Demanding that people wear masks in public is totalitarian.	○	○	○	○	○	○	○

Beliefs in Conspiracy Theories (BICT) Matrix

Please specify how much you agree with each of the below statements

	Strongly disagree	Disagree	Somewhat disagree	Neither agree nor disagree	Somewhat agree	Agree	Strongly agree

	Strongly disagree	Disagree	Somewhat disagree	Neither agree nor disagree	Somewhat agree	Agree	Strongly agree
There was no conspiracy involved in the assassination of John. F. Kennedy.	○	○	○	○	○	○	○
Covid-19 restrictions are in place for political reasons.	○	○	○	○	○	○	○
The European Union is trying to take control of the United Kingdom.	○	○	○	○	○	○	○
The Covid-19 virus was created in a laboratory.	○	○	○	○	○	○	○

Please specify how much you agree with each of the below statements

	Strongly disagree	Disagree	Somewhat disagree	Neither agree nor disagree	Somewhat agree	Agree	Strongly agree
Princess Diana's death was an accident.	○	○	○	○	○	○	○
Governments are suppressing evidence of the existence of aliens.	○	○	○	○	○	○	○
The AIDS virus was created in a laboratory.	○	○	○	○	○	○	○

	Strongly disagree	Disagree	Somewhat disagree	Neither agree nor disagree	Somewhat agree	Agree	Strongly agree
The attack on the Twin Towers was not a terrorist action but a governmental conspiracy.	○	○	○	○	○	○	○

Please specify how much you agree with each of the below statements

	Strongly disagree	Disagree	Somewhat disagree	Neither agree nor disagree	Somewhat agree	Agree	Strongly agree
Masks cause the spread of Covid-19 not prevent it.	○	○	○	○	○	○	○
The American moon landings were faked.	○	○	○	○	○	○	○
A government exercise was behind the suicide at Jones Town.	○	○	○	○	○	○	○
There is a link between Covid-19 and the installation of 5G.	○	○	○	○	○	○	○

Debrief

Debriefing Sheet

Project title: The implication of COVID-19 conspiracy belief and risk perception on pandemic behaviour

Thank you for taking part in this study. The present research investigated the different risks that people perceive in relation to the COVID-19 pandemic and how these relate to behavior in this context.

We also asked some questions relating to beliefs about alternative/unofficial explanations about COVID-19, in order to examine how these relate to different risk perceptions in this context.

All your data will be anonymous and will not be identifiable. Further, your individual data will not be available to anyone outside the research team, as data will be collated for any subsequent write-ups or publications. We kindly ask you to refrain from talking to others about this study while it is ongoing, as this may affect our results from other participants.

If you would like further information about the study or would like to know about what our findings are when all the data have been collected and analysed, then please contact the researchers via the details below. However, please note your individual results cannot be provided as all data is anonymous after the point of collection.

If taking part in this study has affected you in any way, please consider consulting the following sources for support with any COVID-19 related issues:

UK Government support sources:
https://www.gov.uk/find-coronavirus-support

US CDC sources on coping with stress:
https://www.cdc.gov/coronavirus/2019-ncov/daily-life-coping/managing-stress-anxiety.html

If you have any further queries, please contact the researchers:
Sara Komer - sara.komer@durham.ac.uk
Lilli Baxter – lilli.a.baxter@durham.ac.uk
Alexandros Efstratiou – alexandros.efstratiou@durham.ac.uk
Jack Hughes – jack.p.hughes@durham.ac.uk

Dr Ana Leite – ana.castro-leite@durham.ac.uk (Supervisor)
Dr Milica Vasiljevic – milica.vasiljevic@durham.ac.uk (Supervisor)

Please click next to complete the survey.

Powered by Qualtrics
