# Supplementary material for: The impact of risk perceptions and belief in conspiracy theories on COVID-19 pandemic-related behaviours
Source: PLoS One. 2022 Feb 8;17(2):e0263716. doi: 10.1371/journal.pone.0263716 (PMC8824369; doi:10.1371/journal.pone.0263716)
Supplement: S2 Appendix — (DOCX) [file pone.0263716.s002.docx]

**S2 Appendix**

**Kruskal-Wallis sensitivity analyses**

We perform non-parametric Kruskal-Wallis tests to detect whether there are any risk perception differences between people who contracted COVID-19 or had close ones who had contracted it as an added robustness check. Patterns are identical with the comparisons are made between people who answered “yes”, “no”, or “not sure” to the following questions.

*Have you contracted COVID-19?*

Health risks: *H*(2) = .44, *p* = .804

Economy & liberty risks: *H*(2) = 3.16, *p* = .206

Informational risks: *H*(2) = .008, *p* = .996

*Have any of your family or friends contracted COVID-19?*

Health risks: *H*(2) = 3.36, *p* = .187

Economy & liberty risks: *H*(2) = 3.92, *p* = .141

Informational risks: *H*(2) = 2.85, *p* = .241
