# Supplementary material for: The impact of risk perceptions and belief in conspiracy theories on COVID-19 pandemic-related behaviours
Source: PLoS One. 2022 Feb 8;17(2):e0263716. doi: 10.1371/journal.pone.0263716 (PMC8824369; doi:10.1371/journal.pone.0263716)
Supplement: S3 Appendix — (DOCX) [file pone.0263716.s003.docx]

**S3 Appendix**

**Parallel mediation sensitivity analysis with age as covariate**

Here, we report path coefficients for the same analysis under the section *“Results -> Inferential tests -> Mediation model”* while controlling for age. We once again use the HC3 heteroskedasticity-consistent influence.

The overall variance explained and general pattern of results remains largely the same.

Model summary: *F* (5, 349) = 26.42, *p* < .001, *R^2^* = 0.34.

*Path coefficients*

| Outcome variable | Path variable | *B* | *SE* | *t* | *p* | 95% CI |
| --- | --- | --- | --- | --- | --- | --- |
| Health risks | Conspiracy belief | -.36 | .08 | -4.55 | < .001 | [-.52, -.21] |
|  | Age | -.002 | .004 | -.63 | .53 | [0.01, .005] |
| Economy & liberty risks | Conspiracy belief | .54 | .06 | 8.68 | < .001 | [.42, .66] |
|  | Age | .007 | .003 | 2.05 | .041 | [.000, .01] |
| Informational risks | Conspiracy belief | .66 | .06 | 11.24 | < .001 | [.55, .78] |
|  | Age | .003 | .004 | .79 | .43 | [-.004, .01] |
| Compliant behaviour | Conspiracy belief | .08 | .05 | 1.60 | .11 | [-.02, .18] |
|  | Health risks | .39 | .05 | 8.28 | < .001 | [.30, .48] |
|  | Econ. & liberty risks | -.12 | .05 | -2.38 | .02 | [-.23, -.02] |
|  | Informational risks | -.006 | .04 | -.15 | .88 | [-.09, .08] |
|  | Age | -.000 | .003 | -.07 | .95 | [-.006, .005] |
| Compliant behaviour (total effect) | Conspiracy belief | -.13 | .06 | -2.08 | .039 | [-.25, -.007] |
|  | Age | -.002 | .003 | -.59 | .55 | [-.009, .005] |

Direct effect of Conspiracy belief on Compliant behaviour: *B* = .08, *SE* = .05, *t* = 1.60, *p* = .11, 95% CI: [-.02, .18]

Indirect effects on Compliant behaviours:

Total effect: -.21 (95% CI: [-.31, -.12]

Health risks: -.14 (95% CI: [-.22, -.07]

Econ. & Liberty risks: -.07 (95% CI: [-.12, -.01])

Informational risks: -.004 (95% CI: [-.06, .05])
